# Bone marrow-derived dedifferentiated fat cells exhibit similar phenotype as bone marrow mesenchymal stem cells with high osteogenic differentiation and bone regeneration ability

**DOI:** 10.1186/s13018-023-03678-9

**Published:** 2023-03-11

**Authors:** Hirokatsu Sawada, Tomohiko Kazama, Yuki Nagaoka, Yoshinori Arai, Koichiro Kano, Hiroshi Uei, Yasuaki Tokuhashi, Kazuyoshi Nakanishi, Taro Matsumoto

**Affiliations:** 1 Department of Orthopaedic Surgery, Nihon University School of Medicine, Tokyo, Japan; 2 Division of Cell Regeneration and Transplantation, Department of Functional Morphology, Nihon University School of Medicine, 30-1 Oyaguchi-Kamicho, Itabashi-Ku, Tokyo, 173-8610 Japan; 3 Department of Oral and Maxillofacial Radiology, Nihon University School of Dentistry, Tokyo, Japan; 4 Laboratory of Cell and Tissue Biology, College of Bioresource Sciences, Nihon University, Fujisawa, Japan

**Keywords:** Dedifferentiated fat cells, Mesenchymal stem cells, Adipocytes, Cell therapy, Nonunion bone fracture

## Abstract

**Background:**

Mesenchymal stem cells (MSCs) are known to have different differentiation potential depending on the tissue of origin. Dedifferentiated fat cells (DFATs) are MSC-like multipotent cells that can be prepared from mature adipocytes by ceiling culture method. It is still unknown whether DFATs derived from adipocytes in different tissue showed different phenotype and functional properties. In the present study, we prepared bone marrow (BM)-derived DFATs (BM-DFATs), BM-MSCs, subcutaneous (SC) adipose tissue-derived DFATs (SC-DFATs), and adipose tissue-derived stem cells (ASCs) from donor-matched tissue samples. Then, we compared their phenotypes and multilineage differentiation potential in vitro. We also evaluated in vivo bone regeneration ability of these cells using a mouse femoral fracture model.

**Methods:**

BM-DFATs, SC-DFATs, BM-MSCs, and ASCs were prepared from tissue samples of knee osteoarthritis patients who received total knee arthroplasty. Cell surface antigens, gene expression profile, and in vitro differentiation capacity of these cells were determined. In vivo bone regenerative ability of these cells was evaluated by micro-computed tomography imaging at 28 days after local injection of the cells with peptide hydrogel (PHG) in the femoral fracture model in severe combined immunodeficiency mice.

**Results:**

BM-DFATs were successfully generated at similar efficiency as SC-DFATs. Cell surface antigen and gene expression profiles of BM-DFATs were similar to those of BM-MSCs, whereas these profiles of SC-DFATs were similar to those of ASCs. In vitro differentiation analysis revealed that BM-DFATs and BM-MSCs had higher differentiation tendency toward osteoblasts and lower differentiation tendency toward adipocytes compared to SC-DFATs and ASCs. Transplantation of BM-DFATs and BM-MSCs with PHG enhanced bone mineral density at the injection sites compared to PHG alone in the mouse femoral fracture model.

**Conclusions:**

We showed that phenotypic characteristics of BM-DFATs were similar to those of BM-MSCs. BM-DFATs exhibited higher osteogenic differentiation potential and bone regenerative ability compared to SC-DFATs and ASCs. These results suggest that BM-DFATs may be suitable sources of cell-based therapies for patients with nonunion bone fracture.

**Supplementary Information:**

The online version contains supplementary material available at 10.1186/s13018-023-03678-9.

## Background

The incidence of osteoporotic fractures increases in the elderly, mirroring their age-related decrease in bone mineral density (BMD). Autologous bone grafts have been frequently used to promote bone fusion in patients with osteoporotic fractures. However, there are problems with autologous bone grafting such as persistent pain at the donor site, infection of bone, and nerve injury that are associated with the bone harvesting surgical procedure [1].

Currently, the clinical utility of stem cell-based therapy has been shown to overcome the limitations of autologous bone grafting. Mesenchymal stem cells (MSCs) are considered to be an attractive cell source for bone tissue engineering. MSCs have the ability to self-renew and differentiate into various cell types such as adipocytes, chondrocytes, and osteoblasts [2]. The osteogenic potential of MSCs has already been applied in clinical situations such as fracture nonunion, osteogenesis imperfecta, and osteoarthritis [3, 4]. MSCs were originally isolated from bone marrow, but they also can be isolated from other connective tissues such as adipose tissue, umbilical cord, and placenta. MSCs isolated from adipose tissue are referred to as adipose tissue-derived stem cells (ASCs). ASCs have several advantages for clinical application compared to bone marrow MSCs, such as relatively larger stem cell population in the tissue and less invasiveness during collection. MSCs are known to have different differentiation potential depending on the tissue of origin. A previous study using donor-matched MSCs found that bone marrow MSCs showed higher osteogenic and chondrogenic capacity but less adipogenic capacity compared to ASCs [5, 6]. The number of MSCs in tissue and their proliferative ability is reduced according to their donor’s age [7–10]. In addition, MSCs isolated from osteoporosis patients exhibit low proliferative activity and low osteogenic differentiation ability [11, 12]. Therefore, an alternative cell source that can be easily isolated and expanded to adequate amounts for transplantation, especially in elderly subjects and osteoporosis patients, is still required.

Dedifferentiated fat cells (DFATs) are MSC-like multipotent cells that can be prepared from mature adipocytes by ceiling culture method. Our research group reported that DFATs have the potential to differentiate into multiple lineages including adipogenic, osteogenic, chondrogenic, muscular, and neurogenic lineages [13–16], and transplantation of DFATs showed therapeutic effects in a variety of animal models for human diseases [17–22]. Comparative analysis of DFATs with induced pluripotent stem cells using DNA microarray has shown that DFATs exhibit gene expression profiles more similar to mesenchymal progenitor cells than embryonic stem cells [23]. Because DFATs can be prepared from smaller amounts of adipose tissue with higher purity compared to ASCs [13, 24], the cells are thought to be well suited for cell-based therapy for a variety of diseases, including osteochondral diseases. It has not yet fully elucidated whether DFATs derived from adipocytes in different adipose tissue showed different phenotype and functional properties.

In the present study, we prepared bone marrow-derived DFATs (BM-DFATs) and MSCs (BM-MSCs), subcutaneous adipose tissue-derived DFATs (SC-DFATs) and ASCs from donor-matched tissue samples. We then compared their phenotypes and multilineage differentiation capacities in vitro. Furthermore, we evaluated the in vivo bone regeneration potential of these cells using a severe combined immunodeficiency (SCID) mouse femoral fracture model.

## Methods

### Preparation of BM-DFATs, SC-DFATs, BM-MSCs, and ASCs

Femur bone marrow and subcutaneous adipose tissue were provided by knee osteoarthritis patients who had undergone total knee arthroplasty at Itabashi Hospital, Nihon University School of Medicine, Tokyo, Japan (*n* = 9, average age 62.2 ± 15.0 years). Patients with idiopathic osteoarthritis of the knee of grade 3 or 4 according to Kellgren–Lawrence criteria were included. Patients with aggressive synovitis were excluded. Informed consent was given before surgery, and all experiments were conducted with the approval of the Nihon University Clinical Research Review Board. Preparation of SC-DFATs was performed according to a previous report by Matsumoto et al. [13]. Briefly, approximately 1 g of adipose tissue was cut into small pieces and digested with 0.1% (weight/volume) collagenase type I solution (Koken, Tokyo, Japan) at 37 °C for 1 h with gentle agitation. After filtration, the floating top layer containing mature adipocytes was collected by centrifugation at 135 g for 3 min. After washing with phosphate-buffered saline (PBS), the cells (5 × 10^4^) were placed in 12.5-cm^2^ culture flasks (NUNC, Roskilde, Denmark) filled completely with Dulbecco’s modified Eagle’s medium (DMEM, Invitrogen, Carlsbad, CA) containing 20% fetal bovine serum (FBS, JRH Biosciences, Lenexa, KS) and incubated at 37 °C in 5% CO_2_. Mature adipocytes floated up and adhered to the top inner ceiling surface of the flask (Ceiling culture). After 7 days, the medium was removed and the flasks were inverted so that the cells were on the bottom. The medium was changed every 3 or 4 days until the cells reached confluency. For passage, the cells were harvested by treating the cells with a trypsin-ethylenediaminetetraacetic acid solution (Invitrogen), following which the cells were seeded in 100-mm dishes at a density of 1 × 10^6^ cells per dish and cultured. ASCs were prepared according to the preparation method of Zuk et al. [25]. Briefly, approximately 1 g of the adipose tissue was treated with collagenase and centrifuged, and then, the sedimented stromal vascular fraction cells were seeded at a density of 1 × 10^5^ cells/cm^2^ and cultured in DMEM containing 10% FBS. For the preparation of BM-DFATs, isolation of mature adipocytes from bone marrow was performed according to the method described previously [26] with slight modification. Briefly, approximately 5 ml of bone marrow fluid was aspirated with a soft cannula in the femoral distal diaphysis and digested with collagenase type I (Koken) at 37 °C for 30 min. After filtration, the floating top layer containing mature adipocytes was collected by centrifugation at 135 g for 3 min. After washing with PBS, the cells were incubated by the ceiling culture method as described above. BM-MSCs were prepared by the method described previously [27]. Briefly, approximately 5 ml of the bone marrow fluid was centrifuged, and the precipitate fraction cells were seeded at a density of 3 × 10^4^ cells/cm^2^ and cultured in DMEM containing 10% FBS. These four cell types were used for experiments within passage 3. The population doubling time was determined at each passage by the formula:

$${\text{Population}}\;{\text{doubling}}\;{\text{time}} = \ln 2/\left[ {\ln \left( {N/N_{0} } \right)/t} \right]$$

where *N* is the cell number at harvest, *N*_*0*_ is the cell number at seeding, and *t* is the culture period in hours.

### Flow cytometry

The immunophenotypes of the BM-DFATs, SC-DFATs, BM-MSCs, ASCs at passage 2 were identified using flow cytometry as previously described [13]. The cells grown to 60% confluence were suspended at a density of 5 × 10^5^ cells per tube and incubated with various anti-human antibodies conjugated with phycoerythrin (PE) or allophycocyanin (APC). The following antibodies were used: anti-CD73-PE, anti-CD90-APC, anti-CD105-PE, anti-CD31-PE, anti-CD45-APC, anti-HLA-DR-PE, anti-CD106-PE, anti-CD54-APC, and anti-CD36-PE (all from BD Biosciences, San Jose, CA). Mouse IgG1-PE, mouse IgG1-APC, mouse IgG2a-PE, mouse IgG2b-APC, and mouse IgM-PE (all from BD Biosciences) were used as negative controls. The fluorescence intensity of the cells was evaluated by a FACSAria flow cytometer (Becton Dickinson, Bedford, NJ), and data were analyzed using FlowJo software (version 10.6.1, FlowJo, Ashland, OR). Positive cells were counted and compared with the signal of corresponding immunoglobulin isotypes. A minimum of 1 × 10^4^ events were recorded for each sample, and analysis was performed at least three separate times for each condition tested.

### DNA microarray

Total RNA was extracted from SC-DFATs, BM-DFATs, ASCs, and BM-MSCs at passage 0 and 1 using an RNeasy Mini Kit (Qiagen, Hilden, Germany) according to the manufacturer’s instructions. Briefly, 1 × 10^7^ cells were lysed and passed through a prefilter to remove DNA, adjusted with an equal volume of 70% ethanol and applied to the RNA column. After washing, RNA was eluted from the column with 100 µl of water. The quality of the extracted RNA was assessed using an Agilent 2100 Bioanalyzer (Agilent Technologies, Santa Clara, CA). Adequate RNA quality was identified with two clear ribosomal peaks (28S and 18S) and low extraneous noise. Labeling of total RNA was performed using the GeneChip™ 3′IVT PLUS Reagent Kit (Affymetrix, Santa Clara, CA) following the manufacturer’s protocol. Briefly, first- and second-strand cDNA were synthesized from 100 ng of total RNA from each cell sample, and in vitro transcription was performed using biotinylated ribonucleotide analogs to generate aRNA. The labeled aRNA was purified from the mixture and measured and fragmented with the GeneChip™ Hybridization, Wash, and Stain Kit (Thermo Fisher Scientific, Waltham, MA). The RNA samples were hybridized to probes using GeneChip™ Human Genome U133 Plus 2.0 Array (Affymetrix) according to the manufacturer’s instructions. Fluorescent images were visualized using a GeneChip Scanner 3000 (Affymetrix). Gene expression data were analyzed using Transcriptome Analysis Console software (version 4.0, Affymetrix) following the software guidelines. An adjusted *p*-value < 0.05 and log-FC ≥  ± 2.0 were set as the cut-off criteria to screen differentially expressed genes (DEGs).

### In vitro* differentiation assay*

The adipogenic and osteogenic differentiation assay was performed as described previously [13]. Briefly, 5 × 10^4^ cells at passage 3 were seeded on 30-mm dishes (BD Falcon, Franklin Lakes, NJ) and cultured in DMEM containing 10% FBS until reaching confluence. For adipogenic differentiation, the cells were cultured in DMEM containing 10% FBS, 1 µM dexamethasone (Sigma-Aldrich, St. Louis, MO), 0.5 mM isobutylmethylxanthine (Sigma-Aldrich), and 1 × insulin-transferrin-selenium-X (ITS; Invitrogen) for one week. The culture medium was changed every three days. After fixing the cells with 4% paraformaldehyde (Wako, Osaka, Japan), they were stained with Oil red O (Sigma-Aldrich) for 15 min. For osteogenic differentiation, the cells were cultured in DMEM containing 10% FBS, 100 nM dexamethasone, 10 mM β-glycerophosphate (Sigma-Aldrich), and 0.05 mM L-ascorbic acid (Sigma-Aldrich) for one week. The culture medium was changed every three days. After fixing the cells with 4% paraformaldehyde, the cells were incubated at 37 °C for 1 h with 0.16% naphthol AS-TR phosphate (Sigma-Aldrich) and 0.8% Fast Blue BB (Wako) dissolved in 0.1 M Tris buffer (pH 9.0) for detection of alkaline phosphatase (ALP) activity. The cells were also stained with 1% alizarin red S (Sigma-Aldrich) for 3 min at room temperature. The samples were observed under a BX51 microscope (Olympus, Tokyo, Japan).

### Real-time reverse transcription-polymerase chain reaction (RT-PCR)

The mRNA expressions of cells were analyzed by real-time RT-PCR using TaqMan™ gene expression assay. Total mRNA was extracted from cells at passage 3 using an RNeasy Mini Kit, and 1 µg total RNA was reverse-transcribed using a High-Capacity cDNA Reverse Transcription Kit (Life Technologies) according to the manufacturer’s instructions. Subsequently, 5 ng cDNA was analyzed by real-time RT-PCR using TaqMan™ Fast Advanced Master Mix (Applied Biosystems, Foster City, CA) and a StepOnePlus Real-Time PCR System (Applied Biosystems). TaqMan™ probes (Life Technologies) for specific genes were as follows: *PPARG* (PPARγ), Hs001115513_m1; *RUNX2*, Hs00231692_m1; *CEBPA* (C/EBPα), Hs00269972_s1; *ALPL* (Alkaline phosphatase), Hs01029144_m1; *SLC2A4* (GLUT4), Hs00168966_m1; *BGLAP* (Osteocalcin), Hs00168966_m1. Expression level of transcripts was normalized to endogenous human 18S ribosomal RNA (4319413E) mRNA levels according to the formulae comparative Ct. Each sample was analyzed in triplicate.

### Laboratory animals

Male SCID mice were purchased from Oriental Yeast Co., Ltd., Tokyo, Japan. The mice were bred in cages maintained in an optimal environment without restriction on eating and drinking. The animal experiments were performed with the approval of the Animal Experiment Committee of Nihon University School of Medicine. Animal breeding and experiments were conducted in accordance with the Animal Experiment Guideline of Nihon University School of Medicine.

### Mouse femoral fracture model

The mouse femoral fracture model was created according to the report by Bonnarens and Einhorn [28]. Under inhalation anesthesia with isoflurane, a left transverse femoral fracture was created at 10-mm distance from the knee joint using a micro-bone saw (Zimmer Biomet, Warsaw, IN). Then, 50 μl of peptide hydrogel (PHG) Pura Matrix™ (3-D Matrix, Tokyo, Japan) was injected locally into the fracture gap. PHG was prepared according to the manufacturer’s manual. A 25-G injection needle (Terumo, Tokyo, Japan) was inserted intramedullary from the distal femur to fix the bone fracture.

### Time course experiment in the mouse femoral fracture model

Male 8-week-old SCID mice (*n* = 6) were used for the experiment. At 4, 6, and 8 weeks after the femoral fracture, mice were euthanized and both femurs were removed. Micro-computed tomography (CT) images of both femurs were obtained with an R mCT system (Rigaku Co., Ltd., Tokyo, Japan) at 90 kV/100 μA to evaluate the morphological changes of the fracture sites.

### Cell transplantation experiment in the mouse femoral fracture model

Male 8-week-old SCID mice (*n* = 50) were divided into five groups, BM-DFATs group, SC-DFATs group, BM-MSCs group, ASCs group, and Control group, and the fracture model was created for the left femur using the above method (*n* = 10 in each group). After mixing 1 × 10^5^ BM-DFATs, SC-DFATs, BM-MSCs, and ASCs (passage 2) with 50 μl of PHG, the solutions were immediately injected into the fracture gap. In the control group, only 50 μl of PHG was injected into the fracture gap. Four weeks after model creation, all mice were euthanized, and bilateral femurs were removed. Micro-CT images of both femurs were taken to evaluate the effects of transplantation of each cell type on fracture healing. Mice that had an oblique fracture when creating the fracture model were excluded (ASCs group: *n* = 1, Control group: *n* = 1).

### Bone structure analysis

Based on the micro-CT images, bone structure analysis was performed using image analysis software i-viewR (MORITA, Kyoto, Japan). Bone volume (BV) of the femurs was measured in a 4 × 4 × 4 mm^3^ area at the center of the fracture, and BMD was calculated.

### Statistical analysis

All data are expressed as mean ± standard error (SE). For comparison between groups, a test of significant difference was performed by one-way ANOVA and Tukey–Kramer multiple comparison test. A value of *p* < 0.05 was considered as statistically significant. GraphPad Prism Ver5.0 (GraphPad Software, La Jolla, CA) was used for statistical analysis.

## Results

### Phenotypic characteristics of human BM-DFATs, SC-DFATs, BM-MSCs, and ASCs

**Fig. 1:**
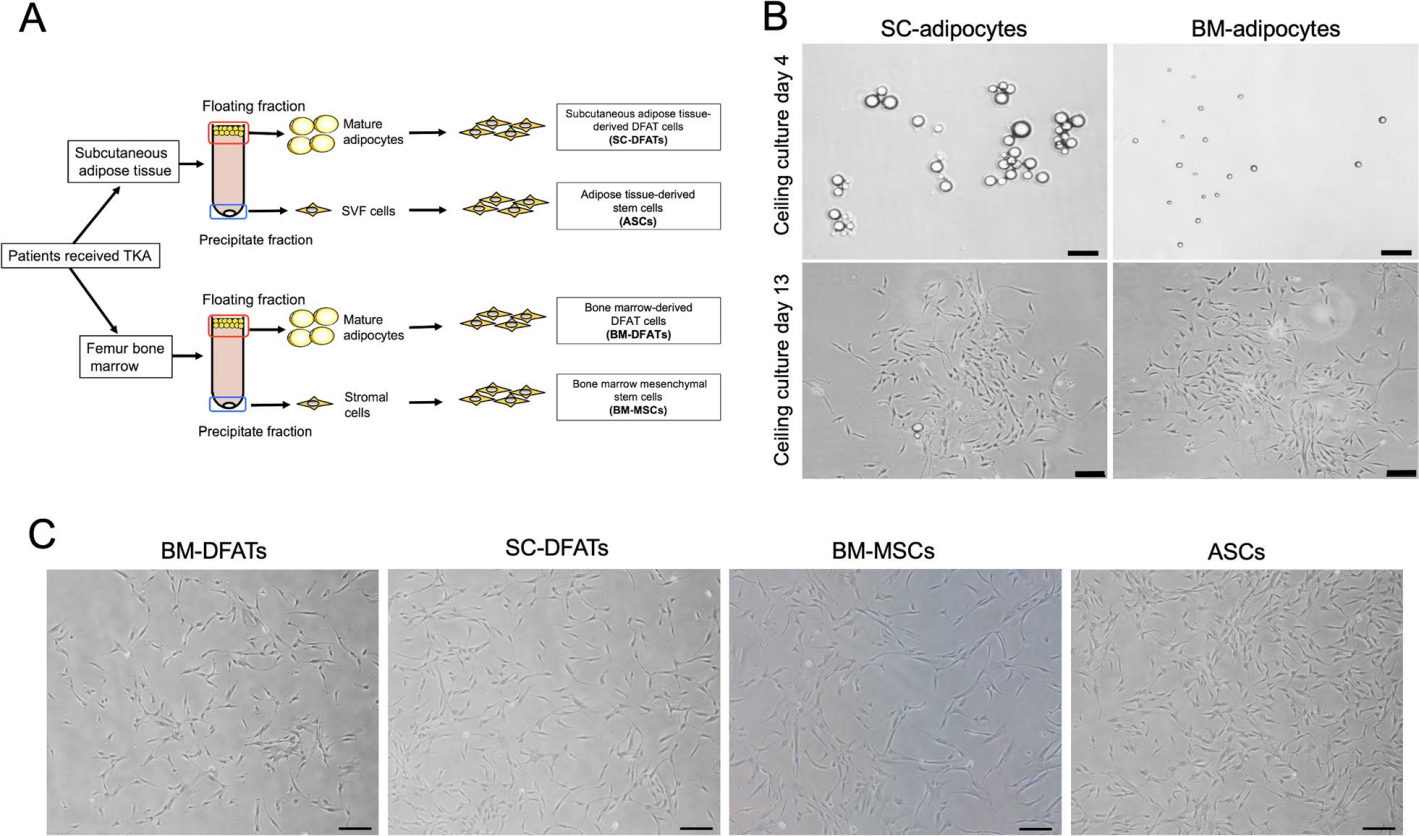
Preparation and morphological analysis of subcutaneous adipose tissue-derived dedifferentiated fat cells (SC-DFATs), adipose tissue-derived stem cells (ASCs), bone marrow-derived dedifferentiated fat cells (BM-DFATs), and bone marrow mesenchymal stem cells (BM-MSCs). **A** Schematic illustration of preparation methods for SC-DFATs, ASCs, BM-DFATs, and BM-MSCs. **B** Representative photomicrographs of adipocytes isolated from subcutaneous adipose tissue and bone marrow at days 4 and 13 after the ceiling culture. Scale bars represent 100 µm. **C** Representative cellular morphology of BM-DFATs, SC-DFATs, BM-MSCs, and ASCs. Scale bars represent 100 μm. TKA: total knee arthroplasty, SVF: stromal vascular fraction

**Fig. 2:**
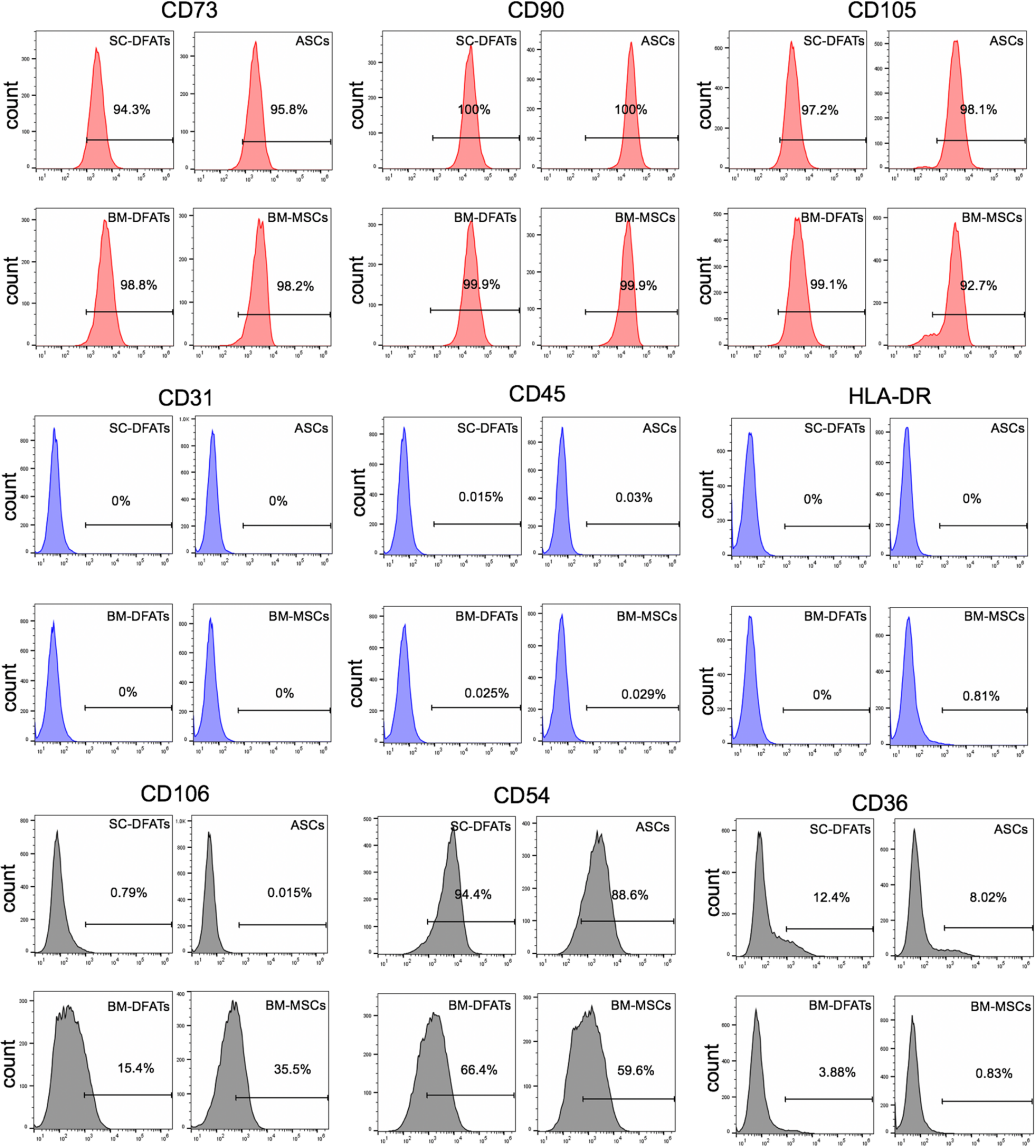
Flow cytometric analysis of SC-DFATs, ASCs, BM-DFATs, and BM-MSCs. Cell surface antigens profiles of SC-DFATs, ASCs, BM-DFATs, and BM-MSCs were determined by flow cytometric analysis. Data are representative of at least three experiments

We first prepared BM-DFATs, SC-DFATs, BM-MSCs, and ASCs from patients with osteoarthritis and examined the phenotypic characteristics of these cell types. Schematic illustration of the preparation methods for these four cell types is shown in Fig. 1A. We confirmed that DFATs could be successfully prepared from mature adipocytes isolated from bone marrow aspirates as well as subcutaneous adipose tissue at day 13 of the ceiling culture, although the size of the mature adipocytes in bone marrow aspirates was much smaller than that in the subcutaneous adipose tissue (Fig. 1B). Morphological analysis revealed that the BM-DFATs, SC-DFATs, BM-MSCs, and ASCs cells exhibited similar spindle-shaped fibroblast-like morphology (Fig. 1C). These four cell types could be prepared reproducibly from all 9 donors examined. The cell growth rate did not differ between the 4 cell types, and the population doubling time was approximately 65 h. These cells could be subcultured for over 8 passages. Flow cytometric analysis showed that the BM-DFATs, SC-DFATs, BM-MSCs, and ASCs expressed MSC markers CD73, CD90, and CD105 at similar levels (Fig. 2). These cells did not express lymphocyte marker CD45, endothelial cell marker CD31, and immunogenic marker HLA-DR, which are known as negative markers for MSCs. HLA-DR^+^ cells were undetected in BM-DFATs, SC-DFATs, and ASCs, whereas they were slightly (0.81%) detected in BM-MSCs. The expression of CD106, known as vascular cell adhesion molecule-1 (VCAM-1), was detected in BM-DFATs (15.4%) and BM-MSCs (35.5%), whereas it was almost non-detectable in SC-DFATs (0.79%) and ASCs (0.015%). The expression of CD54, known as intracellular adhesion molecule 1 (ICAM-1), was frequently observed in SC-DFATs (94.4%) and ASCs (88.6%) compared to that in BM-DFATs (66.4%) and BM-MSCs (59.6%). Similarly, the expression frequency of CD36 in SC-DFATs (12.4%) and ASCs (8.02%) was higher than that in BM-DFATs (3.88%) and BM-MSCs (0.83%). These findings indicate that the BM-DFATs, SC-DFATs, BM-MSCs, and ASCs showed similar morphology and immunophenotype corresponding to the MSC definition, although their immunophenotypes are slightly different in their derived tissue-specific manner.

### Gene expression profiles of BM-DFATs, SC-DFATs, BM-MSCs, and ASCs

**Fig. 3:**
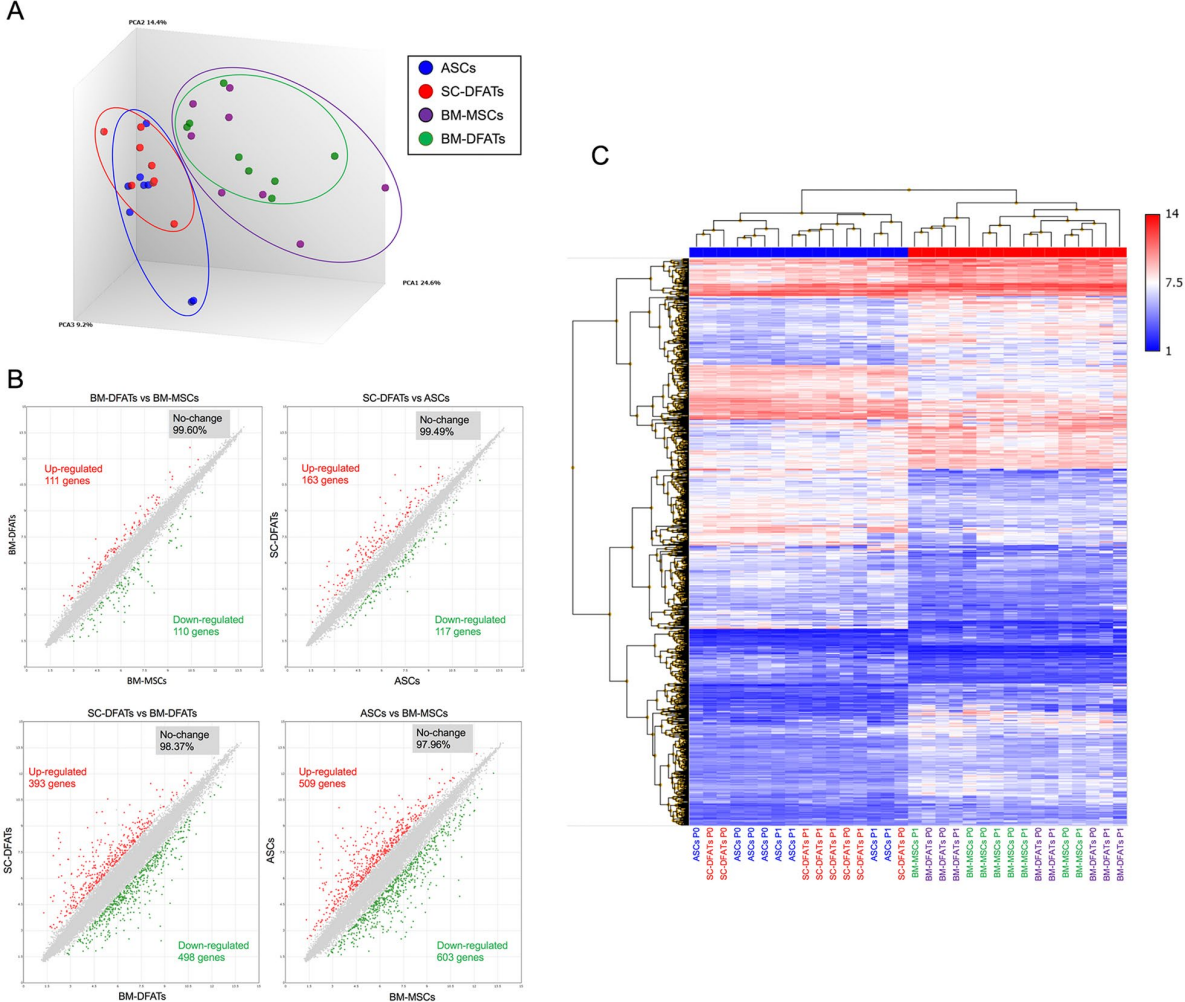
Microarray analysis of SC-DFATs, ASCs, BM-DFATs, and BM-MSCs. Microarray analysis was performed to show differentially expressed genes between SC-DFATs, ASCs, BM-DFATs, and BM-MSCs. **A** Principal component analysis (PCA). **B** Scatter plot analysis. **C** Heat map analysis

We next examined the gene expression profiles of the four cell types prepared from 4 donors using microarray analysis. Principal component analysis revealed that DEGs in SC-derived cells (ASCs and SC-DFATs) were clearly separated from those in BM-derived cells (BM-MSCs and BM-DFATs) (Fig. 3A). Scatter plot analysis confirmed similar expression levels of most genes between BM-DFATs and BM-MSCs (99.6% identical) and between SC-DFATs and ASCs (99.49% identical) (Fig. 3B). Heatmap analysis showed that gene expression profiles in SC-DFATs and ASCs were grouped in a same cluster and clearly separated from those in BM-DFATs and BM-MSCs, regardless of their passage numbers (Fig. 3C). The significantly up-regulated and down-regulated DEGs in BM-DFATs compared with SC-DFATs are listed in Additional file 1: Table S1, with fold change ranging from − 10 to 10. These findings suggest that there are derived tissue-associated differences in gene expression in DFATs and MSCs.

### In vitro* differentiation ability in BM-DFATs, SC-DFATs, BM-MSCs, and ASCs*

**Fig. 4:**
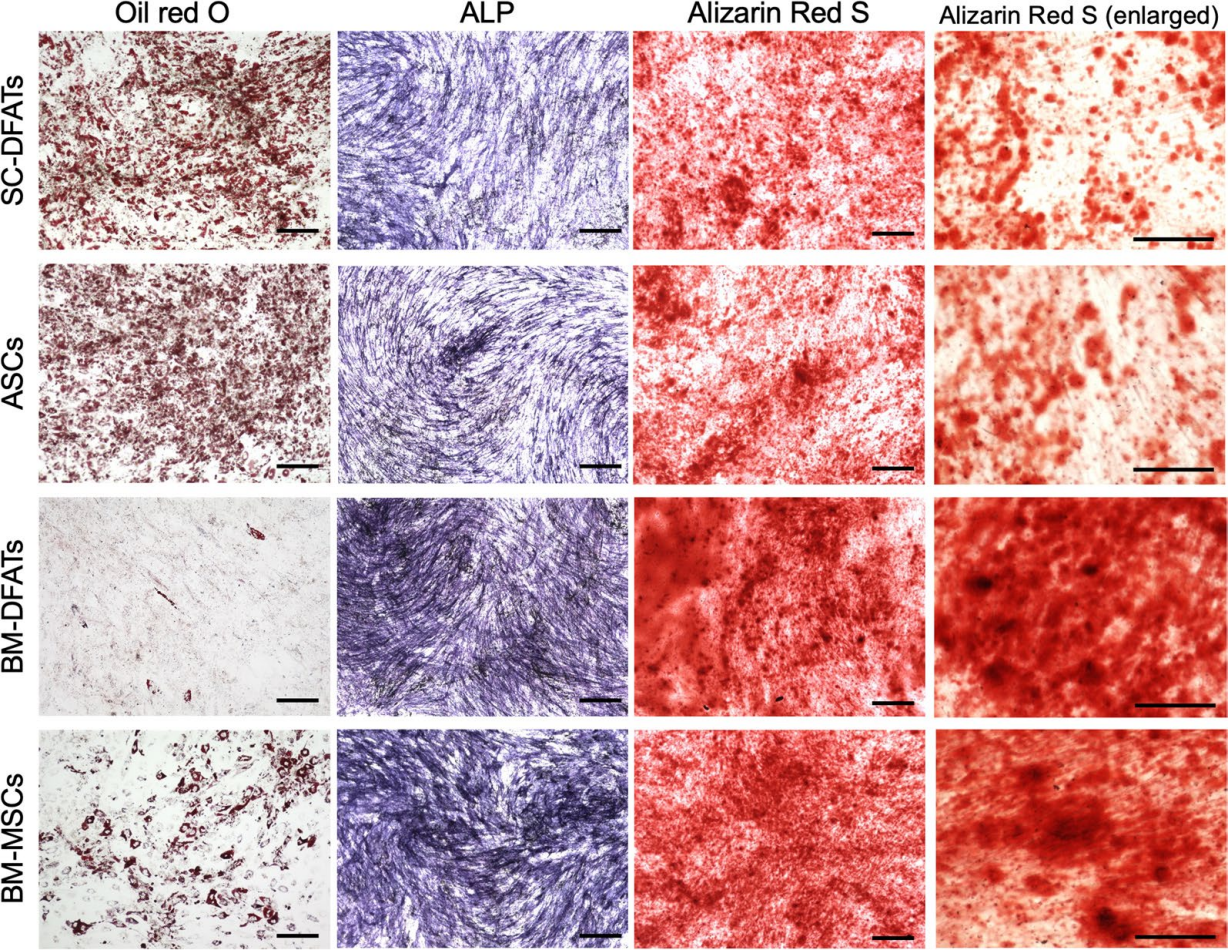
Comparison of in vitro differentiation ability in SC-DFATs, ASCs, BM-DFATs, and BM-MSCs. Representative photomicrographs of Oil red O, alkaline phosphatase (ALP), and alizarin red S staining in SC-DFATs, ASCs, BM-DFATs, and BM-MSCs after 1 week of adipogenic or osteogenic differentiation culture. Scale bars represent 200 μm

We next performed in vitro differentiation assays to clarify the differences of multilineage differentiation ability in these cell types prepared from 4 donors. In the adipogenic differentiation culture, we found that Oil red O-positive lipid-filled adipocytes were observed in the four cell types, although the degrees of lipid droplet deposition in SC-DFATs and ASCs were greater than those in BM-DFATs and BM-MSCs (Fig. 4). In the osteogenic differentiation culture, we found that ALP activity and calcium deposition detected by alizarin red S staining were observed in the four cell types. The intensities of ALP staining in BM-DFATs and BM-MSCs tended to be higher than those in SC-DFATs and ASCs. The alizarin red S staining revealed that larger and thicker calcium deposition was observed in BM-DFATs and BM-MSCs compared to SC-DFATs and ASCs. Similar results were obtained from samples of three other donors.

**Fig. 5:**
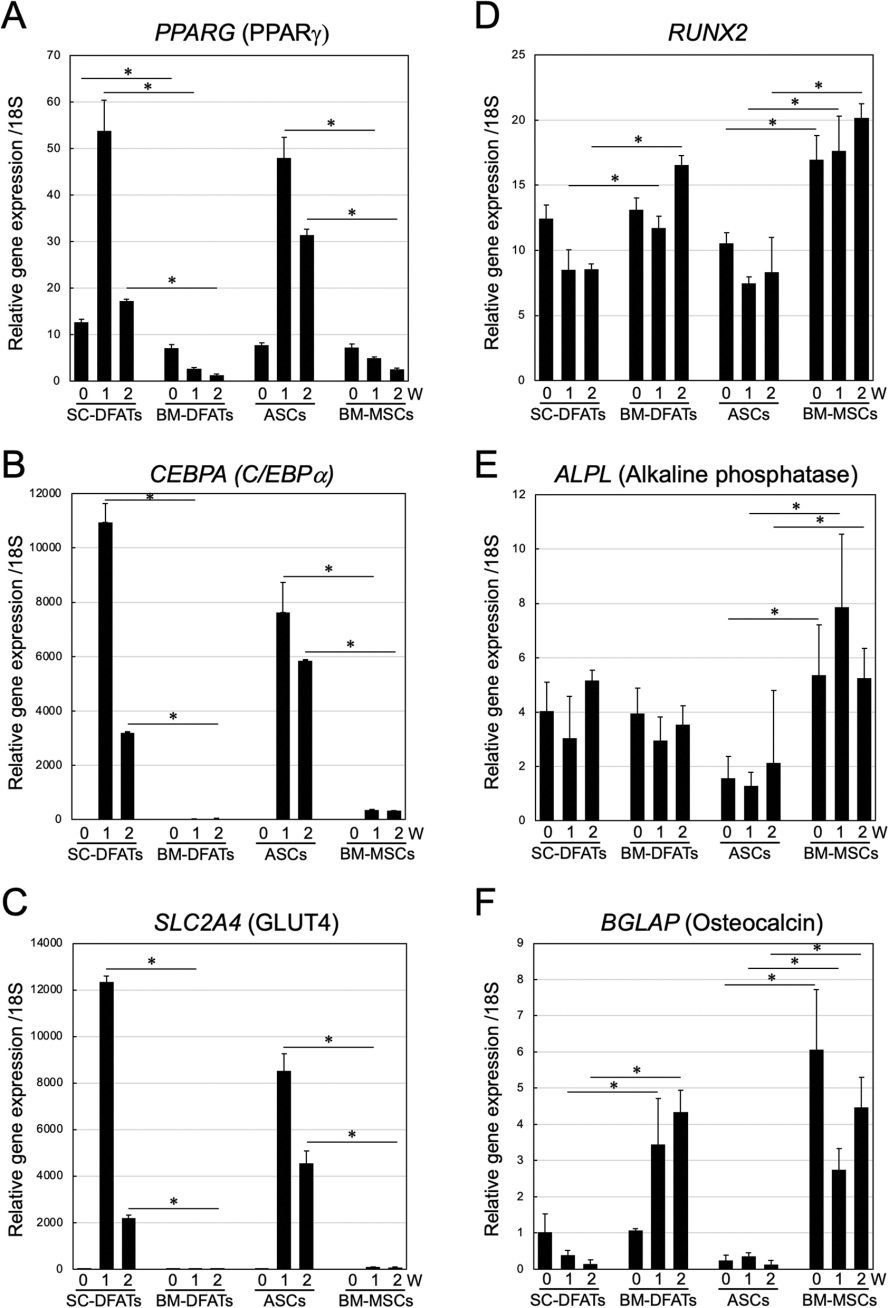
Comparison of gene expression changes in SC-DFATs, ASCs, BM-DFATs, and BM-MSCs. SC-DFATs, ASCs, BM-DFATs, and BM-MSCs were cultured in osteogenic or adipogenic differentiation medium for 2 weeks. Total RNA was extracted at indicated time periods, and real-time RT-PCR analysis was performed. **A-C** Expression of adipogenic marker genes after adipogenic differentiation culture. Expressions of *PPARG* (**A**), *CEBPA* (**B**), and *SLC2A4* (**C**) were evaluated. **D–F** Expression of osteogenic marker genes after osteogenic differentiation culture. Expressions of *RUNX2* (**D)**, *ALPL* **(E)**, and *BGLAP* **(F)** were evaluated. *p < 0.05 (one-way ANOVA, Tukey’s multiple comparison test)

To support these findings, expression of adipogenic marker genes such as *PPARG* (PPARγ), *CEBPA* (C/EBPα), and *SLC2A4* (GLUT4) in SC-DFATs and ASCs were significantly (*p* < 0.05) higher than those in BM-DFATs and BM-MSCs under adipogenic differentiation culture condition (Fig. 5A–C). In contrast, expressions of osteogenic marker genes such as *RUNX2, ALPL* (Alkaline phosphatase), and *BGLAP* (Osteocalcin) in BM-DFATs and BM-MSCs were significantly (*p* < 0.05) higher than those in SC-DFATs and ASCs under the osteogenic differentiation culture condition (Fig. 5D–F). These results indicated that BM-DFATs and BM-MSCs have higher osteogenic differentiation capacity and lower adipogenic differentiation capacity in vitro compared to SC-DFATs and ASCs.

### The bone regenerative effect of BM-DFAT, SC-DFAT, BM-MSC, and ASC transplantation in the mouse femoral fracture model

**Fig. 6:**
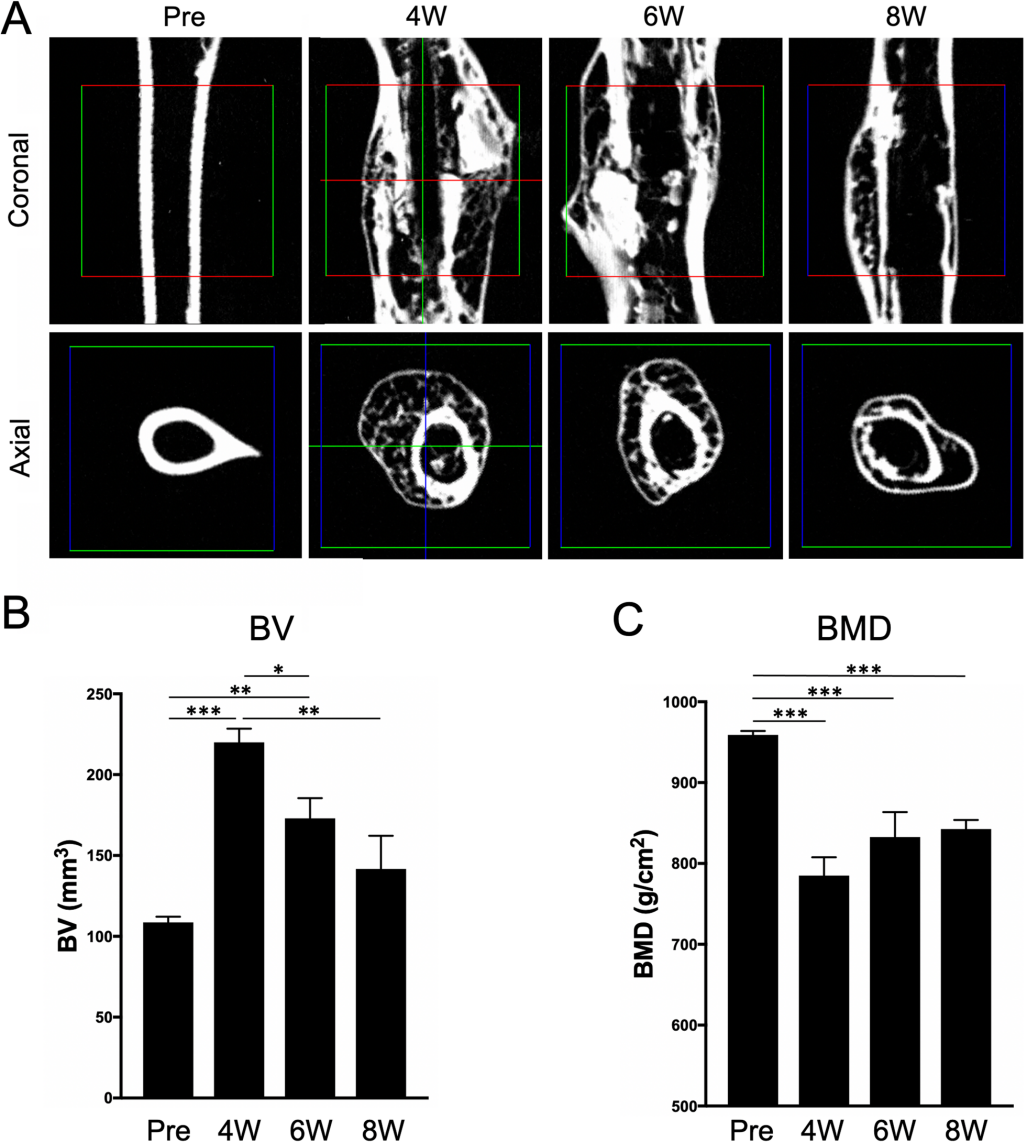
Time-course changes of bone microarchitecture at fracture sites in a mouse femoral fracture model. **A** Representative micro-computed tomography images in coronal and axial views of fractured femurs are shown. The bone volume (BV) (**B**) and the bone mineral density (BMD) (**C**) of the fracture sites were quantified before (Pre) and at 4, 6, and 8 weeks after the femoral fracture. Bars indicate mean ± SE. *p < 0.05, **p < 0.01, ***p < 0.001 (one-way ANOVA, Tukey–Kramer multiple comparison test)

To determine the optimal cell source for bone fracture healing, we next performed in vivo cell transplantation experiments using the mouse femoral fracture model. In this experiment, we used a femoral fracture model in which natural fusion and repair occur after creation of transverse cut in the femur [28]. We first examined time-course changes in bone structure after PHG injection into the fracture gap in this model. PHG is a self-assemble synthetic peptide that acts as a surrogate extracellular matrix to improve tissue regeneration in diverse tissue including bone [29]. The micro-CT images revealed that bone union and remarkable bony callus formation were observed at 4 weeks after the fracture (Fig. 6A). After that, the bony callus was gradually resorbed at 6 and 8 weeks. The bone structure analysis showed that BV at the center of the fracture site, a parameter of bony callus formation, was significantly (*p* < 0.001) increased by over twofold at 4 weeks of treatment compared to pretreatment (Fig. 6B). Then, the BV level gradually decreased at 6 and 8 weeks. The BMD at the fracture callus was significantly (*p* < 0.001) decreased at 4 weeks after the fracture compared to pretreatment followed by a gradual increase in the level with time (Fig. 6C). These results indicated that bone union and bony callus formation occurred by 4 weeks after the fracture, and bone remodeling began after that in this fracture model.

**Fig. 7:**
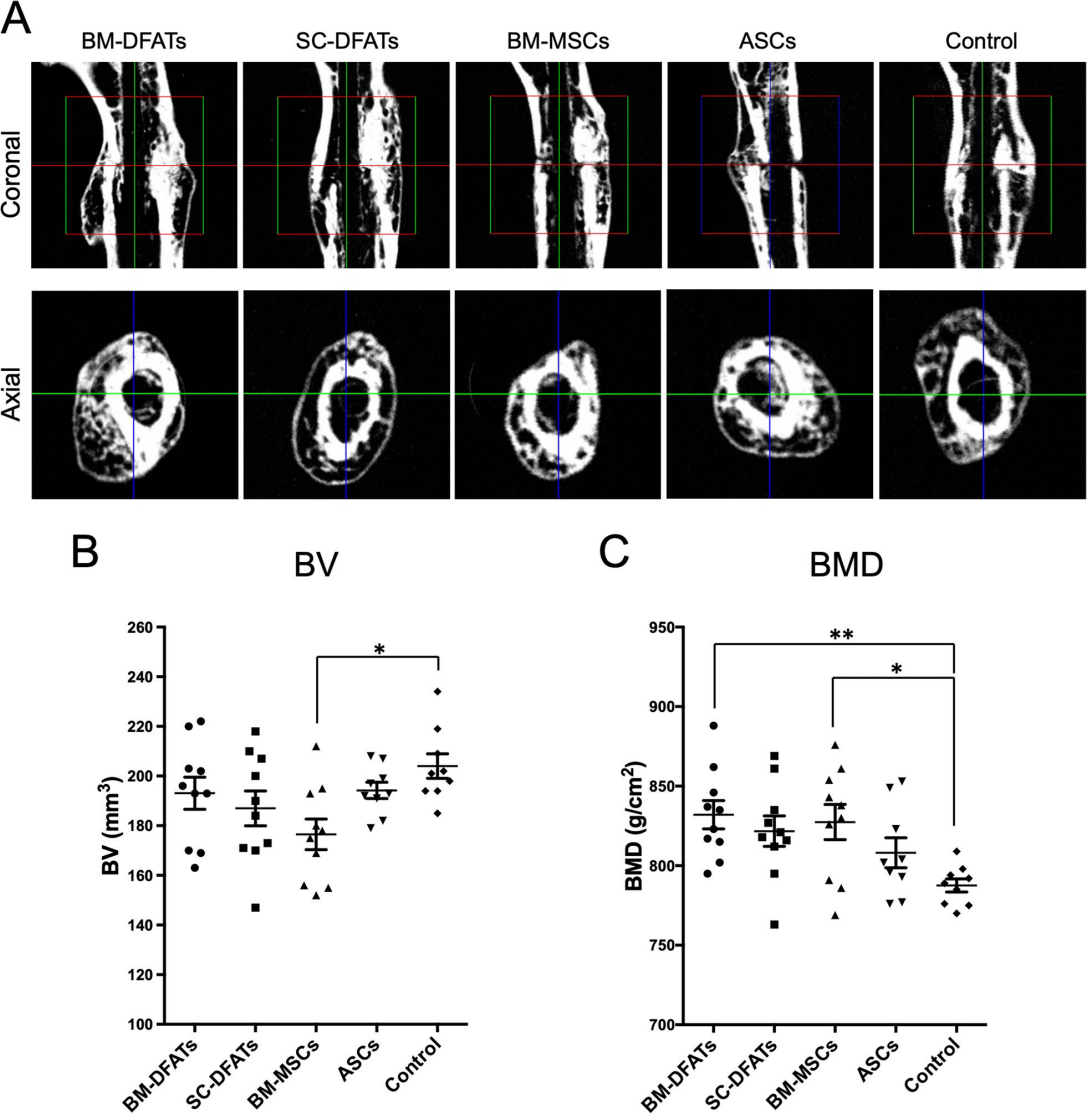
Comparison of bone regenerative effects of transplantation of SC-DFATs, ASCs, BM-DFATs, and BM-MSCs on bone volume (BV) and bone mineral density (BMD) at fracture sites. **A** Representative micro-computed tomography images of fracture sites in each group. **B** Comparison of BV at fracture sites between the 5 groups at 4 weeks after treatment. **C** Comparison of BMD at fracture sites between the 5 groups at 4 weeks after treatment. Bars indicate mean ± SE. *p < 0.05, **p < 0.01 (one-way ANOVA, Tukey–Kramer multiple comparison test)

To investigate in vivo bone regenerative ability of the BM-DFATs, SC-DFATs, BM-MSCs, and ASCs, we next transplanted these four cell types prepared from a 48-year-old donor with PHG in the femoral fracture site and evaluated BV and BMD at 28 days after treatment. The results showed that bone union and bony callus formation were observed in all mice in each group (Fig. 7A). The axial images at the center of the fracture site revealed that new cortical bone thickness and cancellous BMD in the BM-DFATs and BM-MSCs groups tended to be greater compared to those in the control group (PHG alone). Bone structure analysis revealed that the BV in the transplantation groups of all four cell types tended to be lower compared to that in the control group (Fig. 7B). In particular, the BV levels in the BM-MSCs group were significantly (*p* < 0.05) lower than those in the control group. The BMD values in the transplantation groups of all four cell types tended to be higher compared to that in the control group (Fig. 7C), and those in the BM-DFATs group and BM-MSCs group were significantly (*p* < 0.05) higher. These results suggested that transplantation of BM-DFATs and BM-MSCs with PHG enhanced BMD at the injection sites compared to PHG alone in the mouse femoral fracture model.

## Discussion

In this study, we showed that phenotypic features of BM-DFATs are similar to those of BM-MSCs. In addition, the osteogenic differentiation potential and bone regenerative ability of BM-DFATs were greater than those of SC-DFATs and ASCs and were equivalent to BM-MSCs. To our knowledge, the present study is the first to show the phenotypic and functional differences between SC-DFATs and BM-DFATs. We successfully generated BM-DFATs from mature adipocytes in bone marrow aspirates by the conventional ceiling culture method. Although the size of the mature adipocytes in the bone marrow aspirates was much smaller than that in subcutaneous adipose tissue, the BM-DFATs generated showed similar fibroblast-like morphology and immunophenotype similar to SC-DFATs, which is consistent with the definition of MSCs [30]. In the support of a previous study [31], our data confirmed that BM-MSCs expressed higher amounts of CD106 (VCAM-1) and lower amounts of CD54 (ICAM-1) and CD36 compared to ASCs. Interestingly, the expression profiles of these markers in BM-DFATs and SC-DFATs were similar to those in BM-MSCs and ASCs, respectively. In addition, our microarray analysis revealed that the global gene expression profile of BM-DFATs is similar to that of BM-MSCs, whereas the gene expression profile of SC-DFATs is similar to that of ASCs. These findings suggest that there are differences in phenotypic characteristics between DFATs and MSCs that are related to their tissues of origin. Pizzute et al. [32] showed that there are site-dependent phenotypic and functional differences in MSCs. For example, MSCs isolated from bone marrow, adipose tissue, synovial tissue, and muscle tissue tend to differentiate into bone, fat, cartilage, and muscle, respectively.

Our results showed that BM-DFATs had higher osteogenic differentiation potential than SC-DFATs and ASCs. Interestingly, Tsurumachi et al. [33] reported that DFATs prepared from buccal fat pads have higher bone differentiation ability than SC-DFATs. Furthermore, we recently found that DFATs prepared from infrapatellar fat pads have a higher chondrogenic differentiation potential than SC-DFATs prepared from donor-matched samples [34]. These findings suggest that the differentiation tendency of DFATs differs among various fat depots in which the mature adipocytes originated. Notably, DFATs tended to differentiate into the neighboring mesenchymal tissues where their fat depots resided. To support this, Lee et al. [35] reported that adipogenic progenitors localized in different fat depots are intrinsically different, and they may be programmed through epigenetic modulation early during their development. Further studies are needed to explore the mechanisms that give rise to their fat depot-specific phenotypic and functional differences.

BM-MSCs are expected to use tissue engineering and regenerative medicine for patients with refractory bone fracture, and several clinical trials using BM-MSCs are ongoing [36]. MSCs have a low immunogenicity and are widely used not only autologous transplantation but also for allogenic transplantation. However, recent studies showed that allogenic MSCs have limited long-term benefits due to major histocompatibility complex class II upregulation after transplantation, which increases immunogenicity [37, 38]. In the cell replacement therapy for musculoskeletal disorders including refractory bone fracture, autologous MSCs may be more advantageous than allogenic MSCs because they can provide long-term viable cells that can be integrated into patient tissues that are no longer able to repair themselves [39]. However, it is known that the number of BM-MSCs decreases in accordance with a donor’s age and underlying condition such as osteoporosis [40, 41]. In contrast, it is possible to take a sufficient number of mature adipocytes to prepare DFATs by using bone marrow removed during arthroplasty, even in elderly patients. In addition, we found that the proliferative and multilineage differentiation ability of DFATs is not affected by donor age and underlying diseases [13]. These characteristics suggest that BM-DFATs may be a potential cell source for autologous cell therapy in patients with refractory bone fracture that occurs mainly in the elderly.

There are several limitations in this study. First, we studied only cells derived from osteoarthritis patients. It would be desirable to prepare the four cell types from bone marrow and fat of healthy subjects and compare them with cells derived from patients with osteochondral diseases. Second, we did not examine therapeutic effects of each cell type in the femoral fracture model. Further studies such as bone mechanical testing are needed. Third, we evaluated the bone structure change over a short period of time (4 weeks) because the femoral fracture model used in this study naturally recovers within 8 weeks. Long-term effects of cell transplantation should be evaluated using intractable bone fracture model animals.

## Conclusion

The phenotypic characteristics of BM-DFATs were similar to those of BM-MSCs but different from those of SC-DFATs and ASCs. The osteogenic differentiation potential of BM-DFATs was higher than that of SC-DFATs and ASCs and was equivalent to that of BM-MSCs. Transplantation of BM-DFATs and BM-MSCs with PHG increased BMD compared to PHG alone in the mouse femoral fracture model. These results suggest that BM-DFATs may be suitable sources of cell-based therapies for patients with nonunion bone fracture, similar to BM-MSCs.

## Supplementary Information

**Additional file 1: Table S1.** Identification of the up- and down-regulated differentially expressed genes in BM-DFATs compared with SC-DFATs.

## Data Availability

Please contact the corresponding author for data requests.

## Abbreviations

ALP: Alkaline phosphatase
*ALPL*: Alkaline phosphatase (gene name)
APC: Allophycocyanin
ASCs: Adipose tissue-derived stem cells
A*SL2A4* (GLUT4): Glucose transporter type 4
BGLAP (Osteocalcin): Bone gamma-carboxyglutamate protein
BMD: Bone mineral density
BM-DFATs: Bone marrow-derived dedifferentiated fat cells
BM-MSCs: Bone marrow mesenchymal stem cells
BV: Bone volume
*CEBPA* (C/EBPα): CCAAT enhancer-binding protein alpha
CD: Cluster of differentiation
CT: Computed tomography
DEGs: Differentially expressed genes
DFATs: Dedifferentiated fat cells
DMEM: Dulbecco’s modified Eagle’s medium
FBS: Fetal bovine serum
HLA-DR: Human leukocyte antigens with DR specificity
ICAM-1: Intercellular adhesion molecule 1
PE: Phycoerythrin
PHG: Peptide hydrogel
*PPARG* (PPARγ): Peroxisome proliferator-activated receptor γ
RT-PCR: Reverse transcription polymerase chain reaction
SC-DFATs: Subcutaneous adipose tissue-derived dedifferentiated fat cells
SCID: Severe combined immunodeficiency
VCAM-1: Vascular cell adhesion molecule 1

## References

[1] Schmidt AH (2021). Autologous bone graft: Is it still the gold standard?. Injury.

[2] Uccelli A, Moretta L, Pistoia V (2008). Mesenchymal stem cells in health and disease. Nat Rev Immunol.

[3] Zhang ZY, Teoh SH, Hui JH, Fisk NM, Choolani M, Chan JK (2012). The potential of human fetal mesenchymal stem cells for off-the-shelf bone tissue engineering application. Biomaterials.

[4] Grayson WL, Bunnell BA, Martin E, Frazier T, Hung BP, Gimble JM (2015). Stromal cells and stem cells in clinical bone regeneration. Nat Rev Endocrinol.

[5] Mohamed-Ahmed S, Fristad I, Lie SA, Suliman S, Mustafa K, Vindenes H (2018). Adipose-derived and bone marrow mesenchymal stem cells: a donor-matched comparison. Stem Cell Res Ther.

[6] Xu L, Liu Y, Sun Y, Wang B, Xiong Y, Lin W (2017). Tissue source determines the differentiation potentials of mesenchymal stem cells: a comparative study of human mesenchymal stem cells from bone marrow and adipose tissue. Stem Cell Res Ther.

[7] Sethe S, Scutt A, Stolzing A (2006). Aging of mesenchymal stem cells. Ageing Res Rev.

[8] Phinney DG, Kopen G, Righter W, Webster S, Tremain N, Prockop DJ (1999). Donor variation in the growth properties and osteogenic potential of human marrow stromal cells. J Cell Biochem.

[9] Beane OS, Fonseca VC, Cooper LL, Koren G, Darling EM (2014). Impact of aging on the regenerative properties of bone marrow-, muscle-, and adipose-derived mesenchymal stem/stromal cells. PLoS ONE.

[10] Zhou S, Greenberger JS, Epperly MW, Goff JP, Adler C, Leboff MS (2008). Age-related intrinsic changes in human bone-marrow-derived mesenchymal stem cells and their differentiation to osteoblasts. Aging Cell.

[11] Rodriguez JP, Astudillo P, Rios S, Pino AM (2008). Involvement of adipogenic potential of human bone marrow mesenchymal stem cells (MSCs) in osteoporosis. Curr Stem Cell Res Ther.

[12] Rodriguez JP, Montecinos L, Rios S, Reyes P, Martinez J (2000). Mesenchymal stem cells from osteoporotic patients produce a type I collagen-deficient extracellular matrix favoring adipogenic differentiation. J Cell Biochem.

[13] Matsumoto T, Kano K, Kondo D, Fukuda N, Iribe Y, Tanaka N (2008). Mature adipocyte-derived dedifferentiated fat cells exhibit multilineage potential. J Cell Physiol.

[14] Jumabay M, Matsumoto T, Yokoyama S, Kano K, Kusumi Y, Masuko T (2009). Dedifferentiated fat cells convert to cardiomyocyte phenotype and repair infarcted cardiac tissue in rats. J Mol Cell Cardiol.

[15] Sakuma T, Matsumoto T, Kano K, Fukuda N, Obinata D, Yamaguchi K (2009). Mature, adipocyte derived, dedifferentiated fat cells can differentiate into smooth muscle-like cells and contribute to bladder tissue regeneration. J Urol.

[16] Nakano R, Kitanaka T, Namba S, Kitanaka N, Sato M, Shibukawa Y (2020). All-trans retinoic acid induces reprogramming of canine dedifferentiated cells into neuron-like cells. PLoS ONE.

[17] Akita D, Kano K, Saito-Tamura Y, Mashimo T, Sato-Shionome M, Tsurumachi N (2016). Use of rat mature adipocyte-derived dedifferentiated fat cells as a cell source for periodontal tissue regeneration. Front Physiol.

[18] Ikado Y, Obinata D, Matsumoto T, Murata Y, Kano K, Fukuda N (2016). Transplantation of mature adipocyte-derived dedifferentiated fat cells for the treatment of vesicoureteral reflux in a rat model. Int Urol Nephrol.

[19] Obinata D, Matsumoto T, Ikado Y, Sakuma T, Kano K, Fukuda N (2011). Transplantation of mature adipocyte-derived dedifferentiated fat (DFAT) cells improves urethral sphincter contractility in a rat model. Int J Urol.

[20] Kikuta S, Tanaka N, Kazama T, Kazama M, Kano K, Ryu J (2013). Osteogenic effects of dedifferentiated fat cell transplantation in rabbit models of bone defect and ovariectomy-induced osteoporosis. Tissue Eng Part A.

[21] Shimizu M, Matsumoto T, Kikuta S, Ohtaki M, Kano K, Taniguchi H (2018). Transplantation of dedifferentiated fat cell-derived micromass pellets contributed to cartilage repair in the rat osteochondral defect model. J Orthop Sci.

[22] Watanabe H, Goto S, Kato R, Komiyama S, Nagaoka Y, Kazama T (2020). The neovascularization effect of dedifferentiated fat cells. Sci Rep.

[23] Ono H, Oki Y, Bono H, Kano K (2011). Gene expression profiling in multipotent DFAT cells derived from mature adipocytes. Biochem Biophys Res Commun.

[24] Kono S, Kazama T, Kano K, Harada K, Uechi M, Matsumoto T (2014). Phenotypic and functional properties of feline dedifferentiated fat cells and adipose-derived stem cells. Vet J.

[25] Zuk PA, Zhu M, Ashjian P, De Ugarte DA, Huang JI, Mizuno H (2002). Human adipose tissue is a source of multipotent stem cells. Mol Biol Cell.

[26] Attane C, Esteve D, Chaoui K, Iacovoni JS, Corre J, Moutahir M (2020). Human bone marrow is comprised of adipocytes with specific lipid metabolism. Cell Rep.

[27] Pittenger MF, Mackay AM, Beck SC, Jaiswal RK, Douglas R, Mosca JD (1999). Multilineage potential of adult human mesenchymal stem cells. Science.

[28] Bonnarens F, Einhorn TA (1984). Production of a standard closed fracture in laboratory animal bone. J Orthop Res.

[29] Sankar S, O'Neill K, Bagot D'Arc M, Rebeca F, Buffier M, Aleksi E (2021). Clinical use of the self-assembling peptide RADA16: a review of current and future trends in biomedicine. Front Bioeng Biotechnol.

[30] Dominici M, Le Blanc K, Mueller I, Slaper-Cortenbach I, Marini F, Krause D (2006). Minimal criteria for defining multipotent mesenchymal stromal cells. The International Society for Cellular Therapy position statement. Cytotherapy.

[31] Bourin P, Bunnell BA, Casteilla L, Dominici M, Katz AJ, March KL (2013). Stromal cells from the adipose tissue-derived stromal vascular fraction and culture expanded adipose tissue-derived stromal/stem cells: a joint statement of the International Federation for Adipose Therapeutics and Science (IFATS) and the International Society for Cellular Therapy (ISCT). Cytotherapy.

[32] Pizzute T, Lynch K, Pei M (2015). Impact of tissue-specific stem cells on lineage-specific differentiation: a focus on the musculoskeletal system. Stem Cell Rev Rep.

[33] Tsurumachi N, Akita D, Kano K, Matsumoto T, Toriumi T, Kazama T (2016). Small buccal fat pad cells have high osteogenic differentiation potential. Tissue Eng Part C Methods.

[34] Tanimoto K, Matsumoto T, Nagaoka Y, Kazama T, Yamamoto C, Kano K (2022). Phenotypic and functional properties of dedifferentiated fat cells derived from infrapatellar fat pad. Regen Ther..

[35] Lee MJ, Wu Y, Fried SK (2013). Adipose tissue heterogeneity: implication of depot differences in adipose tissue for obesity complications. Mol Aspects Med.

[36] Arthur A, Gronthos S (2020). Clinical application of bone marrow mesenchymal stem/stromal cells to repair skeletal tissue. Int J Mol Sci.

[37] Huang XP, Sun Z, Miyagi Y, McDonald Kinkaid H, Zhang L, Weisel RD (2010). Differentiation of allogeneic mesenchymal stem cells induces immunogenicity and limits their long-term benefits for myocardial repair. Circulation.

[38] Shen S, Li Y, Jin M, Fan D, Pan R, Lin A (2022). CD4(+) CTLs act as a key effector population for allograft rejection of MSCs in a donor MHC-II dependent manner in injured liver. Aging Dis.

[39] Furia JP, Lundeen MA, Hurd JL, Pearce DA, Alt C, Alt EU (2022). Why and how to use the body's own stem cells for regeneration in musculoskeletal disorders: a primer. J Orthop Surg Res.

[40] Muschler GF, Nitto H, Boehm CA, Easley KA (2001). Age- and gender-related changes in the cellularity of human bone marrow and the prevalence of osteoblastic progenitors. J Orthop Res.

[41] Moerman EJ, Teng K, Lipschitz DA, Lecka-Czernik B (2004). Aging activates adipogenic and suppresses osteogenic programs in mesenchymal marrow stroma/stem cells: the role of PPAR-gamma2 transcription factor and TGF-beta/BMP signaling pathways. Aging Cell.

