# Supplementary material for: Bone marrow-derived dedifferentiated fat cells exhibit similar phenotype as bone marrow mesenchymal stem cells with high osteogenic differentiation and bone regeneration ability
Source: J Orthop Surg Res. 2023 Mar 11;18:191. doi: 10.1186/s13018-023-03678-9 (PMC10007822; doi:10.1186/s13018-023-03678-9)
Supplement: Supplementary file 1 — Additional file 1: Table S1. Identification of the up- and down-regulated differentially expressed genes in BM-DFATs compared with SC-DFATs. [file 13018_2023_3678_MOESM1_ESM.docx]

**Supplementary Table 1.** Identification of the up- and down-regulated differentially expressed genes in BM-DFATs compared with SC-DFATs.

| Gene Symbol | Description | Fold Change | P-value | FDR P-value |
| --- | --- | --- | --- | --- |
| Up-regulated in BM-DFATs | | | | |
| FMO3 | flavin containing monooxygenase 3 | 84.79 | 1.35E-16 | 7.00E-13 |
| MAB21L2 | mab-21-like 2 (C. elegans) | 38.36 | 1.37E-21 | 2.50E-17 |
| IGFBP2 | insulin like growth factor binding protein 2 | 28.47 | 3.01E-13 | 4.44E-10 |
| COL14A1 | collagen, type XIV, alpha 1 | 19.43 | 0.0002 | 0.0055 |
| CRISPLD1 | cysteine-rich secretory protein LCCL domain containing 1 | 17.93 | 1.85E-15 | 5.95E-12 |
| TGFB2 | transforming growth factor beta 2 | 14.9 | 1.02E-13 | 1.86E-10 |
| KRTAP1-1 | keratin associated protein 1-1 | 12.87 | 2.06E-06 | 0.0002 |
| ANKRD1 | ankyrin repeat domain 1 (cardiac muscle) | 12.77 | 4.58E-09 | 1.51E-06 |
| VCAM1 | vascular cell adhesion molecule 1 | 11.34 | 1.39E-11 | 1.35E-08 |
| C11orf87 | chromosome 11 open reading frame 87 | 10.25 | 7.62E-14 | 1.44E-10 |
| C5orf46 | chromosome 5 open reading frame 46 | 10.25 | 1.24E-08 | 3.39E-06 |
| Down-regulated in BM-DFATs | | | | |
| EPB41L3 | erythrocyte membrane protein band 4.1-like 3 | -73.27 | 6.24E-15 | 1.54E-11 |
| CNTN3 | contactin 3 (plasmacytoma associated) | -42.18 | 4.90E-21 | 6.69E-17 |
| MEOX2 | mesenchyme homeobox 2 | -40.45 | 7.68E-17 | 5.24E-13 |
| CLDN11 | claudin 11 | -26.87 | 1.29E-06 | 0.0001 |
| ZFPM2 | zinc finger protein, FOG family member 2 | -24.43 | 9.78E-27 | 5.34E-22 |
| CD36 | CD36 molecule (thrombospondin receptor) | -23.79 | 6.94E-08 | 1.33E-05 |
| IL13RA2 | interleukin 13 receptor, alpha 2 | -21.96 | 6.13E-08 | 1.21E-05 |
| MMP3 | matrix metallopeptidase 3 | -19.59 | 4.62E-05 | 0.0019 |
| EMX2 | empty spiracles homeobox 2 | -19.54 | 1.41E-16 | 7.00E-13 |
| EBF2 | early B-cell factor 2 | -15.85 | 3.66E-14 | 7.15E-11 |
| LAMA2 | laminin, alpha 2 | -15.07 | 1.21E-20 | 1.33E-16 |
| ITM2A | integral membrane protein 2A | -15 | 1.24E-16 | 7.00E-13 |
| S100A4 | S100 calcium binding protein A4 | -13.9 | 1.13E-08 | 3.15E-06 |
| MME | membrane metallo-endopeptidase | -13.88 | 6.54E-05 | 0.0024 |
| SERPINB2 | serpin peptidase inhibitor, clade B (ovalbumin), member 2 | -13.45 | 5.70E-08 | 1.15E-05 |
| SFRP1 | secreted frizzled-related protein 1 | -12.32 | 1.38E-09 | 5.76E-07 |
| KCND2 | potassium channel, voltage gated Shal related subfamily D, member 2 | -12.3 | 7.93E-16 | 3.09E-12 |
| MOXD1 | monooxygenase, DBH-like 1 | -10.77 | 1.29E-08 | 3.44E-06 |
| XG | Xg blood group | -10.73 | 1.97E-10 | 1.24E-07 |
| CLEC2B | C-type lectin domain family 2, member B | -10.63 | 6.47E-15 | 1.54E-11 |
| ZNF521 | zinc finger protein 521 | -10.47 | 1.05E-05 | 0.0006 |

# The inclusion criteria for the differentially expressed genes are following: up-regulated genes must have a log2 fold change ≥ 10 and adjusted p-value < 0.05, while down-regulated genes must have a log2 fold change ≤ -10 and adjusted p-value < 0.05.
